# Understanding the Role and Value of Process Quality Indicators in Hospitalized Older Surgical Patients

**DOI:** 10.1093/geroni/igab046.2235

**Published:** 2021-12-17

**Authors:** Janani Thillainadesan, Sarah Hilmer, Alison Mudge, Sarah Aitken, Vasi Naganathan

**Affiliations:** 1 Concord Hospital, NSW, New South Wales, Australia; 2 The University of Sydney, St Leonards, New South Wales, Australia; 3 Royal Brisbane and Women’s Hospital, Royal Brisbane and Women’s Hospital, Queensland, Australia; 4 University of Sydney, Concord, New South Wales, Australia

## Abstract

Background Despite the development of geriatrics surgery process quality indicators (QIs), few studies have reported on these QIs in routine surgical practice. Even less is known about the links between these QIs and clinical outcomes, and patient characteristics. We aimed to measure geriatrics surgery process QIs, and investigate the association between process QIs and outcomes, and QIs and patient characteristics, in hospitalized older vascular surgery patients. Methods This was a prospective cohort study of 150 consecutive patients aged ≥ 65 years admitted to a tertiary vascular surgery unit. Occurrence of geriatrics surgery process QIs as part of routine vascular surgery care was measured. Associations between QIs and high-risk patient characteristics, and QIs and clinical outcomes were assessed using clustered heatmaps. Results QI occurrence rate varied substantially from 2% to 93%. Some QIs, such as cognition and delirium screening, documented treatment preferences, and geriatrician consultation were infrequent and clustered with high-risk patients. There were two major process-outcome clusters: (a) multidisciplinary consultations, communication and screening-based process QIs with multiple adverse outcomes, and (b) documentation and prescribing-related QIs with fewer adverse outcomes. Conclusions Clustering patterns of process QIs with clinical outcomes are complex, and there is a differential occurrence of QIs within older vascular surgery patients, suggesting process QIs alone may be unreliable targets for quality improvement. Prospective intervention studies are needed to understand the causal pathways between process QIs and outcomes to help prioritize care processes that are most clearly linked to improved outcomes.

